# The *Caenorhabditis elegans* CUB-like-domain containing protein RBT-1 functions as a receptor for *Bacillus thuringiensis* Cry6Aa toxin

**DOI:** 10.1371/journal.ppat.1008501

**Published:** 2020-05-05

**Authors:** Jianwei Shi, Donghai Peng, Fengjuan Zhang, Lifang Ruan, Ming Sun

**Affiliations:** State Key Laboratory of Agricultural Microbiology, College of Life Science and Technology, Huazhong Agricultural University, Wuhan, China; University of Massachusetts Medical School, UNITED STATES

## Abstract

Plant-parasitic nematodes cause huge agricultural economic losses. Two major families of *Bacillus thuringiensis* crystal proteins, Cry5 and Cry6, show nematicidal activity. Previous work showed that binding to midgut receptors is a limiting step in Cry toxin mode of action. In the case of Cry5Ba, certain *Caenorhabditis elegans* glycolipids were identified as receptors of this toxin. However, the receptors for Cry6 toxin remain unknown. In this study, the *C*. *elegans* CUB-like-domain containing protein RBT-1, released by phosphatidylinositol-specific phospholipase C (PI-PLC), was identified as a Cry6Aa binding protein by affinity chromatography. RBT-1 contained a predicted glycosylphosphatidylinositol (GPI) anchor site and was shown to locate in lipid rafts in the surface of the midgut cells. Western ligand blot assays and ELISA binding analysis confirmed the binding interaction between Cry6Aa and RBT-1 showing high affinity and specificity. In addition, the mutation of *rbt-1* gene decreased the susceptibility of *C*. *elegans* to Cry6Aa but not that of Cry5Ba. Furthermore, RBT-1 mediated the uptake of Cry6Aa into *C*. *elegans* gut cells, and was shown to be involved in triggering pore-formation activity, indicating that RBT-1 is required for the interaction of Cry6Aa with the nematode midgut cells. These results support that RBT-1 is a functional receptor for Cry6Aa.

## Author summary

*Bacillus thuringiensis* (Bt) crystal proteins belong to pore-forming toxins (PFTs), which display virulence against target hosts by forming holes in the cell membrane. Cry6A is a nematicidal PFT, which exhibits unique protein structure and different mode of action than Cry5B, another nematicidal PFT. However, little is known about the mode of action of Cry6A. Although an intracellular nematicidal necrosis pathway of Cry6A was reported, its extracellular mode of action remains unknown. We here demonstrate that the CUB-like-domain containing protein RBT-1 acts as a functional receptor of Cry6A, which mediates the intestinal cell interaction and nematicidal activity of this toxin. RBT-1 represents a new class of crystal protein receptors. RBT-1 is dispensable for Cry5B toxicity against nematodes, consistent with that Cry6A and Cry5B have different nematicidal mechanisms. We also find that Cry6A kills nematodes by complex mechanism since *rbt-1* mutation did not affect Cry6A-mediated necrosis signaling pathway. This work not only enhances the understanding of Bt crystal protein-nematode mechanism, but is also in favor for the application of Cry6A in nematode control.

## Introduction

*Bacillus thuringiensis* (Bt) is a ubiquitous spore-forming bacterium, which produce a large family of environmentally friendly crystal proteins (Cry), with toxicity against target insects (Lepidoptera, Diptera, Coleoptera, Hymenoptera, Homoptera, Orthoptera, and Mallophaga) or nematodes [1]. Due to its highly and specifically insecticidal activity, *B*. *thuringiensis* has been developed as the leading biopesticide as an alternative to synthetic chemical pesticides [2]. Additionally, some Cry protein genes have also been used to generate transgenic crops for pest control [3]. However, the emergence of insect resistance became a major threat for the long-term use of Bt based biocontrol products [4]. One of the resistance-related mechanisms is linked to mutation of the Cry protein-binding cell surface receptors [5]. Thus, it is critical for insect and nematode biocontrol to identify the receptor genes of Cry proteins in different pests.

The Cry protein receptors participate in binding toxins, leading to membrane insertion, pore formation, cell lysis and death of the hosts [4]. In insects from three different insect orders, similar Cry protein receptors have been reported, such as glycosylphosphatidylinositol (GPI)-anchored protein aminopeptidase-N (APN), GPI-anchored alkaline phosphatase (ALP) and transmembrane protein cadherin [4]. Recently, an ATP-binding cassette transporter in different lepidopteran larvae [6], a sodium solute symporter in *Tribolium castaneum* [7] and an α-amylase in *Anopheles albimanus* [8] have also been identified as Cry protein receptors.

Besides insects, a variety of plant-parasitic nematodes have brought enormous agricultural economic losses (estimated $US80-157 billion) [9, 10]. To date, nematicidal activity has been reported in several *B*. *thuringiensis* Cry protein families, such as Cry5 and Cry6 [11, 12]. Interestingly, Cry6Aa, a novel nematicidal ClyA-type alpha-pore-forming toxin [13–15], does not contain the conserved blocks of classical three-domain (3D) structures, which are present in most of insecticidal Cry proteins and nematicidal Cry5Ba [1, 16]. The free-living soil nematode *Caenorhabditis elegans* has been exploited as a suitable model for studying interaction between hosts and pathogens [17–19]. Some glycolipids have been shown to act as the receptors for Cry5Ba in *C*. *elegans* [20]. Moreover, *C*. *elegans bre* mutants, deficient in glycolipid synthesis, showed to be resistant to Cy5Ba, but not to Cry6Aa [21, 22], indicating that these toxins may have different mode of action.

Although Cry6Aa could kill nematodes by necrosis pathway [23], the cell surface receptors of this toxin remain unknown. Here, we identified a *C*. *elegans* GPI-anchored protein RBT-1, which specifically bound Cry6Aa and was shown to be involved in Cry6Aa intoxication, suggesting that RBT-1 is a functional receptor for Cry6Aa.

## Methods

### Strains

*B*. *thuringiensis* BMB0250 [24], harboring plasmid pBMB0250 encoding Cry6Aa, was cultivated in liquid ICPM medium [25] with erythromycin (25 μg/ml). *Escherichia coli* strains were grown in Luria-Bertani (LB) liquid medium supplemented with ampicillin (100 μg/ml) or kanamycin (50 μg/ml). *C*. *elegans* strains were maintained on nematode-growth media (NGM) plates seeded with *E*. *coli* OP50 at 20°C using standard techniques [26]. *C*. *elegans* strains used include wild-type Bristol strain N2, PHX1304: *rbt-1(syb1304)*, in which 91% of coding sequence (from 4G to 1336A) of *rbt-1* was deleted, and IE3768: *rbt-1(ttTi3768)*, which is a mutant strain with the insertion of *Mos* transposon in the *rbt-1* gene.

### Purification and labeling of Cry6Aa protein

For Cry protein purification, *B*. *thuringiensis* BMB0250 were cultivated in liquid ICPM until the spores and crystals had separated. The crude spore lysate pellets were treated using the method of Griffitts *et al*. [27]. The purified protein samples were then solubilized in 20 mM HEPES (Calbiochem BB0364) (pH 8.0), quantified, and stored at -80°C until used. The purified Cry6Aa protein was respectively labeled with N-hydroxysuccinimide-rhodamine (Pierce 46102) and N-hydroxysulfosuccinimide ester-PC-biotin (Pierce, Rockford, IL), according to the manufacturer’s instructions.

### Precondensation of Triton X-114 and fractionation of GPI-anchored proteins from *C*. *elegans*

Precondensation of Triton X-114 (Sigma-Aldrich, St. Louis, MO) and fractionation of GPI-anchored proteins from *C*. *elegans* was performed according to the described methods [28]. The resulting GPI-anchored protein-enriched fraction was maintained at -80°C until used.

### Pull-down affinity isolation of Cry6Aa binding proteins

Streptavidin-agarose (Sigma-Aldrich) was added to biotinylated-Cry6Aa protein and the sample was incubated for 1 h at 20°C. After centrifugation at 750 × g for 3 min at 20°C, the affinity resin pellet was washed 3 times with TBS. The GPI-anchored protein-enriched fraction obtained from *C*. *elegans* as described above was then incubated with the Cry6Aa-agarose column for 1 h at 20°C. The mixture was centrifuged and the affinity resin pellet was washed 3 times as described above. The bound proteins were then eluted by boiling for 10 min in sample buffer (0.125 M Tris-HCl pH 6.8, 4% SDS, 20% glycerol, 10% dithiothreitol, 0.02% bromophenol blue). After centrifugation at 14,000 × g for 5 min at 20°C, the supernatant was separated by sodium dodecyl sulfate-polyacrylamide gel electrophoresis (SDS-PAGE).

The bands were stained with Coomassie-blue. A strong band of 50 kDa size was clearly observed in the gel of GPI-anchored proteins. This band was excised, trypsin digested, and analyzed by LC-MS/MS.

### Cloning, expression, and purification of recombinant protein

Total RNA from *C*. *elegans* N2 was prepared with *TransZol* Up Plus RNA Kit (TransGen Biotech). cDNA was synthesized from total RNA using *TransScript* One-Step gDNA Removal and cDNA Synthesis SuperMix (TransGen Biotech). The gene that codes for truncated RBT-1 protein devoid of signal peptide and the downstream sequence of the GPI-anchoring site was amplified and cloned into the prokaryotic expression vector pGEX-6P-1. The primers used were F35_1 (CGC GGA TCC GTT GAT TTT AAT TGT CCC G) and F35_3 (CCG CTC GAG TTA ATT ATA TTT CTT GAA TTC). *E*. *coli* BL21 was used to express recombinant proteins. Recombinant *E*. *coli* cells were grown in LB medium up to an A_600_ of 0.6 at 37°C. Protein expression was induced by the addition of 0.1 mM isopropyl β-D-1-thiogalactopyranoside and bacterial cultures were incubated at 16°C overnight. Proteins were purified on GSTrap FF column according to the manufacturer’s instructions (Amersham Biosciences). The elution was dialyzed and stored at -80°C. The CUB-like domain of RBT-1 was expressed by using the same method, and the primers used were F35_1 and F35_2 (CCG CTC GAG TTA AAA TCG ATA AGT GAC CAT G).

To express Cry6Aa in *E*. *coli*, the plasmid pET-28a(+) with gene *cry6Aa* was transformed into BL21 strain. The primers used were Pet-6aF (CCG GAA TTC ATT ATT GAT AGT AAA ACG) and Pet-6aR (CCG GTC GAC TTA ATT ATT ATA CCA ATC CG). Protein expression was induced as described above.

For construction of RNAi strains for silencing *rbt-1* expression, *E*. *coli* strain HT115 was transformed with RNAi plasmid pL4440 containing a gene fragment from *rbt-1* gene. The primers used were F35RNAiF (ATC TGA TAA ATC AAG CTG CCA CG) and F35RNAiR (ATA GTC GAC TGG AAC TAG CGG TGA GTC CT). The dsRNA synthesis was induced as previously described [29].

### Western ligand blot

To analyze the binding interaction between RBT-1 and Cry6Aa, GST-tagged truncated RBT-1, GST and Cry6Aa were electrophoresed in 10% SDS-PAGE, and then the gel was electrotransferred to nitrocellulose membrane (Millipore, Bedford, MA, USA). After blocking with 3% bovine serum albumin (BSA) in phosphate buffered saline (PBS)-T buffer (1% Tween-20 in PBS, pH 7.4), nitrocellulose membrane was incubated in buffer containing 5 ng/ml Cry6Aa for 2 h at room temperature. The bound Cry6Aa was visualized with anti-Cry6Aa antibody (1:5000 dilution) for 2 h at room temperature, followed by incubation with anti-rabbit secondary antibody (1:5000 dilution) coupled to horseradish peroxidase (HRP) for 2 h at room temperature. The membrane was washed after each incubation. Then, the membrane was visualized by using 3,3′-Diaminobenzidine tetrahydrochloride as described by the manufacturers.

### Enzyme-linked immunosorbent assay (ELISA)

ELISA 96-well plates (Costar, NY) were coated overnight at 4°C with 0.5 μg/well of GST-tagged truncated RBT-1 in PBS buffer. Wells were washed with PBS containing 0.05% Tween-20 (PBS-Tween) and incubated for 1 h at room temperature with PBS-Tween containing 3% BSA (PBST-BSA). For the binding assays, biotinylated-Cry6Aa was diluted to different concentrations in PBST-BSA. For the competition assays, a 1000-fold molar excess of unlabeled Cry6Aa was added into the solution containing biotinylated-Cry6Aa. Each well of the ELISA plates was incubated with a 100 μL aliquot of biotinylated-Cry6Aa for 2 h at room temperature and washed three times with PBS-Tween. Then, each well was incubated with 100 μL of HRP-conjugated streptavidin diluted 1:10000 in PBST-BSA (SA-HRP; Pierce) for 1 h at room temperature. After plates were washed as above, each well was incubated with the peroxidase substrate TMB (3,3’,5,5’-tetramethylbenzidine) for 5 min at room temperature. The enzymatic reaction was then stopped with 2 M sulfuric acid, and the absorbance at 450 nm was measured. Specific binding was determined by subtracting non-specific binding from total binding. All assays were performed at least two times. Data were analyzed using SigmaPlot software (Version 11; Systat Software Inc., San Jose, CA), and the curves were fitted based on a best fit of the data to a one-site saturation binding equation [30].

### Nematode bioassays

Nematode bioassays were conducted in 96-well plate with M9 buffer at 25°C, and OP50 was used as source of food. For quantitative growth assays, synchronized L1 worms were added to 96-well plate with Cry6Aa protein at different concentrations, according to the reported method [31]. After 3 days, at least 30 worms for each condition were photographed at ×100 magnification using a compound microscope (Olympus) and the average areas of worms for each protein concentration were normalized to that of the no-toxin control. For mortality assays, synchronized L4 worms were used. After Cry6Aa or Cry5Ba treatment, a visibly moving nematode was marked as alive, and nematodes that failed to respond to several touches were marked as dead. To determine the effect of Cry protein on animals’ development, synchronized L1 worms were added to 20 μg/ml Cry6Aa containing plates and incubated at 20°C for 5 days. By using microscopic examination, the developmental stages of worms were determined by the gonad and body size. As for RNAi plate assay, L1 animals were fed with dsRNA-producing bacteria and grown to L4 stage at 20°C. Then collected worms were transferred to plates with Cry6Aa-expressing *E*. *coli* or control bacteria and incubated for 3 days. As for heat shock assay, synchronized L4 worms were grown at 37°C, and alive worms were recorded every 2 h. For lifespan assay, synchronized L4 nematodes were cultured at 25°C, and alive worms were recorded every day.

### Assays for endocytosis of rhodamine-labeled Cry6Aa

Assays for endocytosis was performed as described [27], wild-type and mutant *C*. *elegans* samples (L4 stage) were respectively incubated in M9 medium containing rhodamine-labeled Cry6Aa (or heat-inactivated Cry6Aa, or nontoxic BSA) of 50 μg/ml. Then animals were sampled after 2 h and washed five times in M9 medium before observation using Olympus fluorescence microscope. Images were captured using ×40 objective lens. Fluorescence was monitored simultaneously with a rhodamine filter.

### Lysosome-like gut granule visualization

Lysotracker (Life Technologies, USA) was used to observe lysosomal morphology [32]. Nematodes were treated with 50 μg/ml Cry6Aa for 6 days and 20 μM Lysotracker for 2 h followed by microscopic examination. The excitation and emission wavelengths were 555 nm and 580 nm.

### Propidium iodide uptake assays

For pore-formation assays, propidium iodide (sigma) uptake assays were performed according to the protocol reported by Kourtis *et al* [33]. Nematodes were incubated with 50 μg/ml Cry6Aa for 4 h and 20 μM propidium iodide for 30 min, and then visualized using a fluorescence microscope. The excitation and emission wavelengths were 555 nm and 580 nm, respectively. Images were processed with ImageJ software.

### Bioinformatic analysis

The signal peptide of RBT-1 was predicted using the program SignalP 4.1 (http://www.cbs.dtu.dk/services/SignalP/) [34]. Prediction of potential C-terminal GPI modification sites was done with big-PI predictor (http://mendel.imp.ac.at/sat/gpi/gpi_server.html) [35]. Possible sites for N-glycosylation were analyzed by the program NetNGlyc 1.0 (http://www.cbs.dtu.dk/services/NetNGlyc/) [36].

## Results

### Identification of RBT-1 as a putative Cry6Aa binding protein

**Fig 1:**
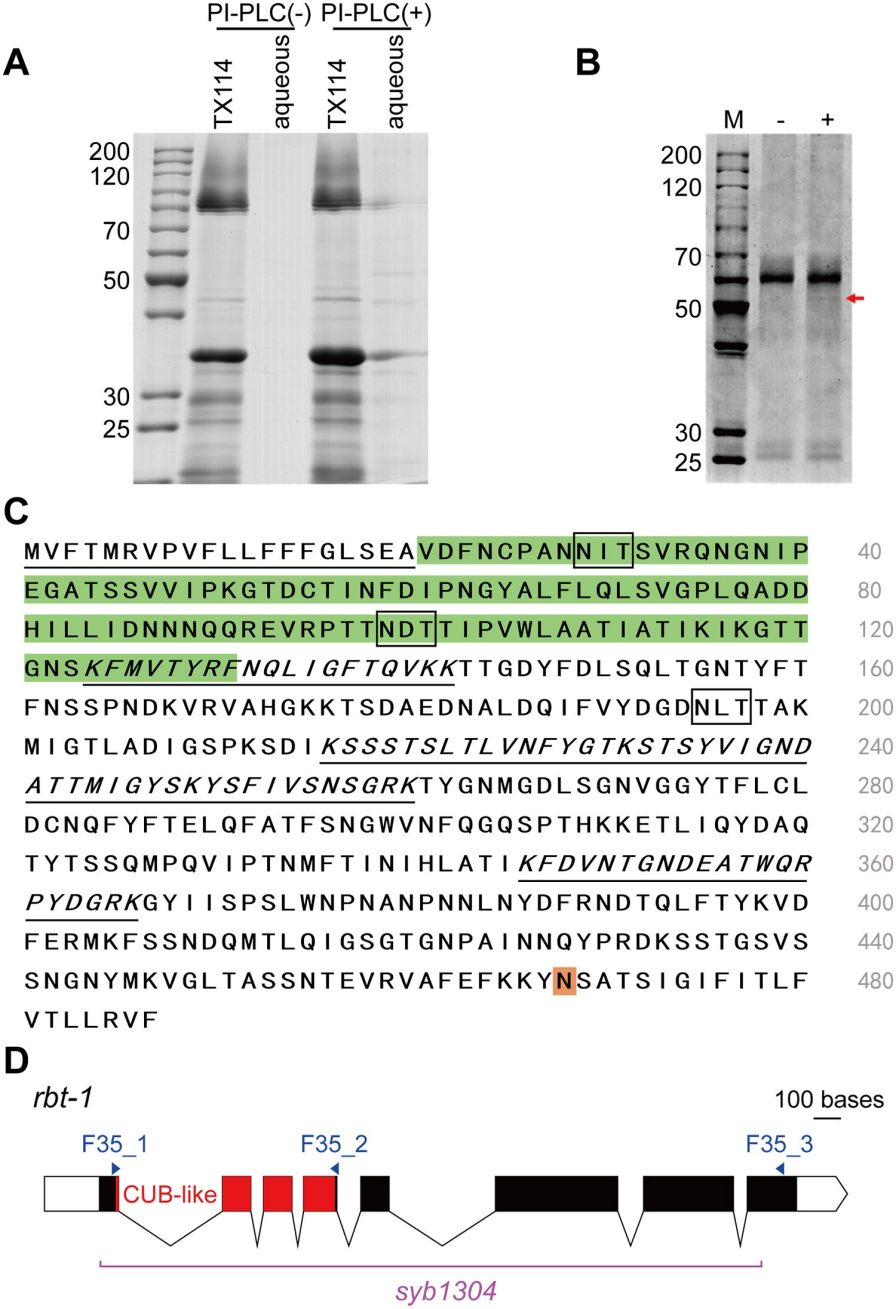
Identification of RBT-1 as a GPI-anchored binding partner for Cry6Aa. (A) The extracted GPI-anchored proteins were analyzed by SDS-PAGE and stained with Coomassie blue. SDS-PAGE of the detergent-rich phase (TX114) and aqueous phase are shown without (PI-PLC (-)) and with PI-PLC (PI-PLC (+)) treatment. (B) Cry6Aa binds a GPI-anchored protein. Streptavidin agarose beads were incubated with the biotinylated Cry6Aa, then the resulting complexes were incubated with the extracted GPI-anchored proteins shown in panel A as aqueous PI-PLC. After washing unbound proteins the sample was then analyzed in SDS-PAGE (+), as control we incubated Cry6Aa coupled beads with TBS (-). The arrow marks the stronger observed band with a size of 50 kDa protein, which was sequenced by LC-MS/MS. (C) The amino acid sequence of RBT-1. The N-terminal hydrophobic domain that corresponds to the putative signal peptide is underlined. The putative C-terminal GPI-anchor site is shaded in orange. The putative N-glycosylation sites are in bold boxes. Peptide sequences obtained from LC-MS/MS identification are in italic and underlined. The CUB-like domain is highlighted in green. (D) Gene model of *rbt-1*, made in the Exon-Intron Graphic Maker (http://wormweb.org/exonintron). Exons (colored boxes) are linked by introns (lines). White boxes represent the 5’ and 3’ UTR and red boxes indicate the protein domain. The purple line points to the deleted sequence in the allele *syb1304*. Arrowheads indicate primers used for amplifying the fragments of RBT-1 and the CUB-like domain. Scale bar, 100 bases.

GPI-anchored proteins are present in germline cells and somatic cells of *C*. *elegans* [37]. We initially isolated GPI-anchored proteins from wild-type *C*. *elegans* N2 by phase partition with the detergent Triton X-114 [28]. GPI-anchored proteins partitioned into the detergent-rich phase were treated with PI-PLC to specifically cleave GPI anchors, followed by the Triton X-114 treatment. The resulting proteins in the aqueous phase after PI-PLC treatment were analyzed by SDS-PAGE (Fig 1A). The aqueous phase without PI-PLC treatment contained few proteins, while the aqueous with PI-PLC treatment revealed several protein bands.

To identify GPI-anchored proteins that potentially interact with Cry6Aa in *C*. *elegans*, we performed pull-down affinity chromatography assays using biotinylated Cry6Aa coupled to streptavidin-agarose. Streptavidin-agarose, conjugated with biotinylated Cry6Aa, was incubated with the GPI-anchored proteins that were extracted from wild-type *C*. *elegans* N2 as described in Methods. After washing unbound proteins, the proteins retained on the Cry6Aa-coupled beads were separated by means of SDS-PAGE. Typical results are shown in Fig 1B. Among several proteins, a protein of approximately 50 kDa specifically bound to the Cry6Aa agarose beads. In the control treatment where GPI-anchored proteins were replaced with TBS buffer, this band was not observed suggesting specific binding to Cry6Aa.

To identify the 50-kDa protein, the band on the Coomassie-stained gel was picked up, digested with trypsin and the resulting fragments were analyzed by LC-MS/MS (Fig 1B). The peptide sequences obtained are shown in Fig 1C. The WormBase database (taxonomic restriction to *C*. *elegans*) and the NCBInr database (taxonomic restriction to *E*. *coli*) was searched with LC-MS/MS data using Mascot to establish the best protein matches. The peptide sequences showed high identity to a single protein identified as F35E12.10 from *C*. *elegans*.

The deduced *f35e12*.*10* gene sequence, called *rbt-1* (for Receptor for *B**acillus thuringiensis* Cry Toxin), is shown in Fig 1D. The deduced amino acid sequence of RBT-1 protein revealed that it is composed of 487 amino acids with a theoretical molecular size of 54 kDa and a pI of 6.9. Analysis of RBT-1 predicts a cleavage site for a putative N-terminal signal peptide between A20 and V21, a C-terminal GPI site with N468 as the predicted ω-site, as well as sites for potential N-glycosylation (N29, N99 and N195) (Fig 1C). All these characteristics agree with the proposed identification of the approximately 50-kDa protein as RBT-1 that binds to Cry6Aa protein. RBT-1 contains a CUB-like domain (Fig 1C and 1D). CUB-containing proteins are always extracellular and plasma membrane-associated proteins, which perform a variety of functions, such as cell signaling and receptor-mediated endocytosis [38].

### Binding interaction of Cry6Aa with RBT-1

**Fig 2:**
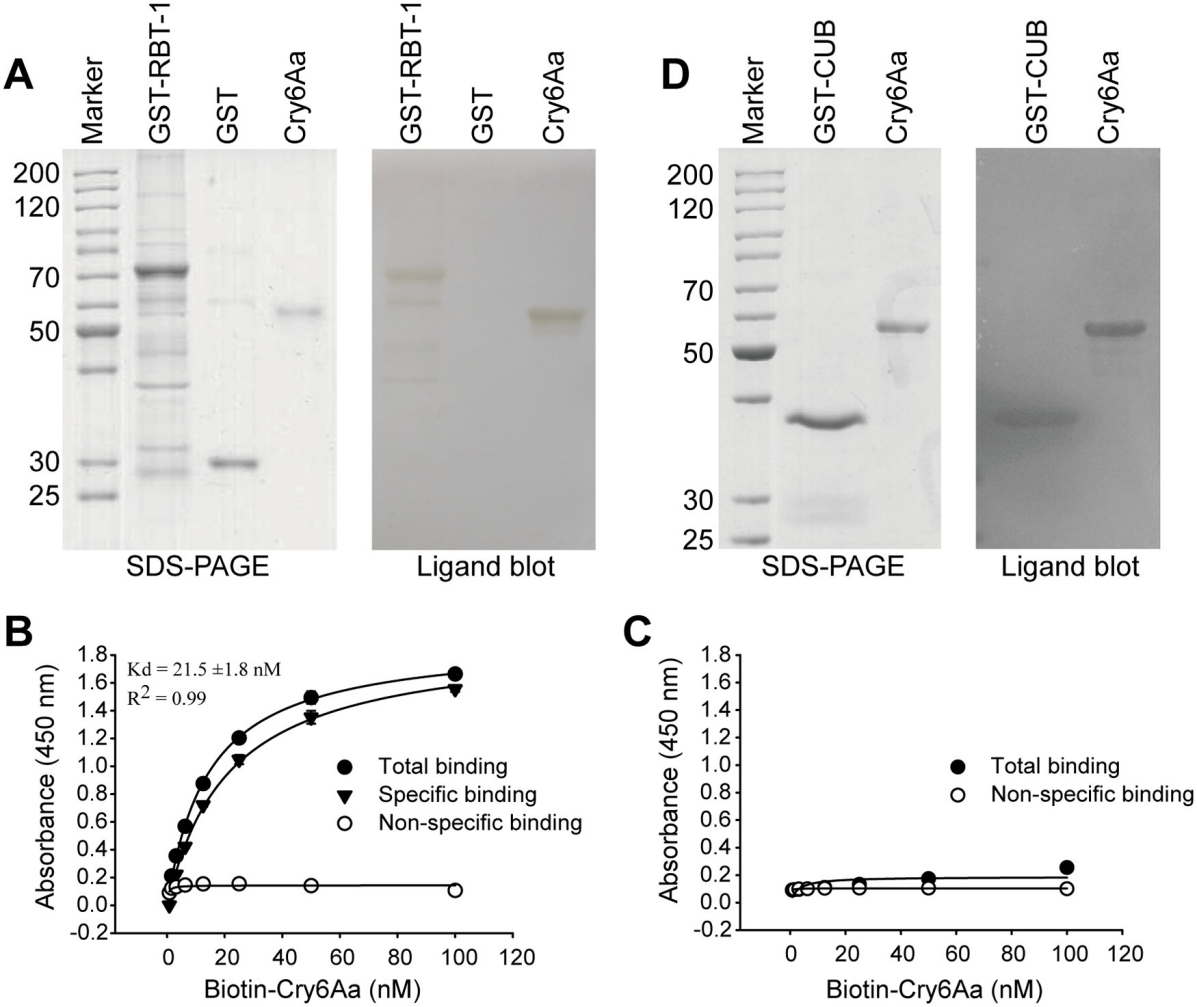
Binding interaction between Cry6Aa and RBT-1. (A) *In vitro* western ligand blot for testing the interaction between Cry6Aa and RBT-1. Purified GST-RBT-1, GST and Cry6Aa were separated on SDS-PAGE and visualized by staining with Coomassie Brilliant Blue R250 (left). An identical gel was electrotransferred to NC membrane, which was incubated with 5 nM Cry6Aa as described in Methods. After removing unbound Cry6Aa, the membrane was incubated with anti-Cry6Aa antibody followed by incubation with secondary antibody coupled to peroxidase (right). (B, C) Binding affinity of GST-RBT-1 (B) or GST (C) to Cry6Aa. ELISA 96-well plates coated with 0.5 μg of GST-RBT-1 or GST were incubated with increasing concentrations of biotinylated Cry6Aa alone or with 1000-fold molar excess of unlabeled Cry6Aa to determine specific binding. Error bars indicate standard deviation. These data were performed in triplicate. (D) Western ligand blot showing the interaction between Cry6Aa and CUB-like domain of RBT-1 performed as described in panel A.

In order to confirm the binding interaction between Cry6Aa and RBT-1, we performed *in vitro* western ligand blot and enzyme-linked immunosorbent assays (ELISA). An RBT-1 protein fragment lacking the putative signal peptide (V21-Y467) was expressed as a glutathione S-transferase (GST) fusion protein (GST-RBT-1) in *E*. *coli*. The predicted molecular weight of GST-RBT-1 is approximately 70 kDa. Purified GST-RBT-1, GST and Cry6Aa proteins were separated by SDS-PAGE (Fig 2A). Additional bands of lower molecular weight were observed in the GST-RBT-1 sample. Binding of Cry6Aa to GST-RBT-1 was performed as described in Methods. Fig 2A shows that GST-RBT-1 bound Cry6Aa. Cry6Aa also bound some of the proteins of lower molecular weight suggesting that these proteins are likely degradation products of the 70 kDa RBT-1 fusion protein (Fig 2A). In contrast, the negative control consisting of the GST protein did not bind to Cry6Aa (Fig 2A).

ELISA binding assays were performed as described in Methods to obtain relative binding affinity of Cry6Aa to RBT-1 under non-denaturing conditions. As shown in Fig 2B and 2C, biotin-labeled Cry6Aa showed dose-dependent binding to RBT-1, but not GST, with a predicted high affinity of 21.5 nM. Meanwhile, the specificity of binding of Cry6Aa to RBT-1 was demonstrated by a homologous competition experiment, since binding of biotin-labeled Cry6Aa to GST-RBT-1was completely competed by 1000-fold excess of unlabeled Cry6Aa (Fig 2B).

Furthermore, because RBT-1 contains a CUB-like domain, we investigated whether this domain was involved in Cry6Aa binding. First, we expressed and purified GST-fused CUB-like domain of RBT-1 (GST-CUB) and separated it by SDS-PAGE. Western ligand blots revealed that GST-CUB binds Cry6Aa protein (Fig 2D).

### *rbt-1* mutation reduces the susceptibility of *C*. *elegans* to Cry6Aa

**Fig 3:**
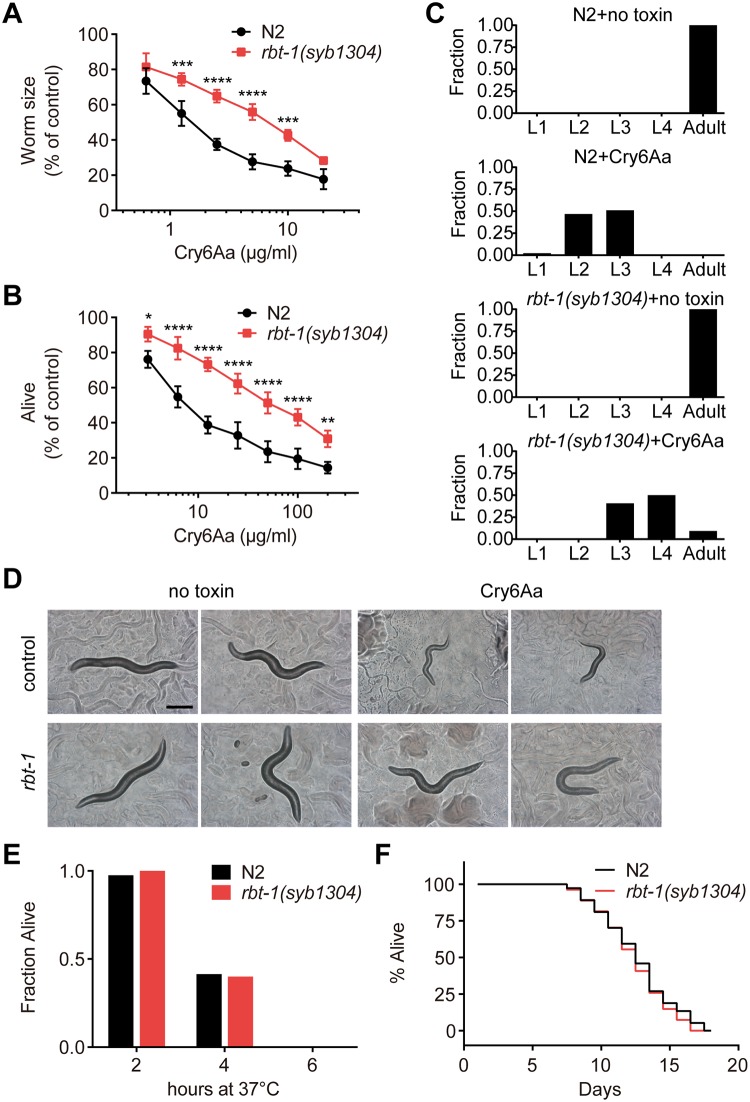
RBT-1 is involved in Cry6Aa toxicity against *C*. *elegans*. Dose-dependent growth assay (A) and mortality assay (B) were performed using Cry6Aa to quantitatively compare the sensitivities of wild-type N2 to mutant *rbt-1(syb1304)* worms. Both assays showed *rbt-1(syb1304)* reduced the susceptibility to Cry6Aa compared with N2. Values are means ± SD (*n* = 3 independent trials). Asterisks represent significant differences (*n* = 100 animals; *P < 0.05, **P < 0.01, ***P < 0.001 and ****P < 0.0001; two-way ANOVA). (C) A Cry6Aa development inhibition assay was performed with synchronized L1 stage worms of wild-type N2 to mutant *rbt-1(syb1304)*. The animals were exposed to 20 μg/ml Cry6Aa. Developmental stages were assessed and presented as the percent of population reaching the indicated development stage after 3 days (without toxin) or 5 days (with Cry6Aa). Approximately 50 animals were used for each strain. (D) Toxicity assays using *E*. *coli*-expressed Cry6Aa on the plate to animals subjected to RNA interference assays. Wild-type N2 treated with RNAi empty vector (pL4440) or dsRNA for gene *rbt-1* were transferred to plates containing the *E*. *coli*-expressed either Cry6Aa (Cry6Aa) or without toxin (no toxin) for 3 days. Reducing *rbt-1* gene expression resulted in the reduced susceptibility to Cry6Aa. Two representative animals for each condition are shown. The experiment was performed in triplicate. Approximately 100 animals were used for each treatment. Scale bar, 200 μm. (E) A heat shock assay was used to compare the thermotolerance of wild-type N2 and mutant *rbt-1(syb1304)*. The experiment was performed in triplicate. No differences were observed. (F) A lifespan assay. *rbt-1(syb1304)* showed similar lifespan to wild-type N2 at 25°C. Median survival was 13 days for both populations N2 and *rbt-1(syb1304)*. Data were obtained from three independent experiments. On average, 30 animals were tested per data point.

It has been shown that mutations of toxin receptors result in resistance, such as *C*. *elegans* mutants that lack glycolipid receptors and are resistant to Cry5Ba [20, 27]. To determine whether RBT-1 participates in the toxicity of Cry6Aa, bioassays were carried out to observe the difference between the wild-type N2 and mutant *rbt-1(syb1304)*. We found that *rbt-1(syb1304)* animals survived better under Cry6Aa treatment compared with N2. During continuous exposure to Cry6Aa, mutant *rbt-1(syb1304)* had statistically greater worm size and higher survival rate than wild-type N2 (Fig 3A and 3B). RBT-1-mediated resistance against Cry6Aa was confirmed in *rbt-1(ttTi3768)* animals which contain a different *rbt-1* mutant allele (S1 Fig). As for the development of Cry6Aa-treated worms, *rbt-1(syb1304)* animals could grow to L4 and adult stages in the presence of Cry6Aa while most N2 were arrested in L2 and L3 stages (Fig 3C). To confirm whether the resistance is due to the mutation of *rbt-1*, we analyzed the susceptibility to Cry6Aa toxin of animals after silencing *rbt-1* expression by RNAi. As shown in Fig 3D, worms fed with dsRNA targeting *rbt-1* showed significant lower susceptibility to *E*. *coli*-expressed Cry6Aa compared with control worms. These results indicate that RBT-1 mediates Cry6Aa toxicity against nematodes. Both N2 and *rbt-1(syb1304)* nematodes grew healthily under normal culture condition and had similar thermotolerance (Fig 3C and 3E), and the mutation of *rbt-1* did not affect worm lifespan (Fig 3F), suggesting that mutant *rbt-1(syb1304)* and N2 have similar growth characteristics and that the resistant phenotype of *rbt-1(syb1304)* is relatively specific for Cry6Aa toxin.

### RBT-1 is involved in the uptake of Cry6Aa into intestinal cells of *C*. *elegans*

**Fig 4:**
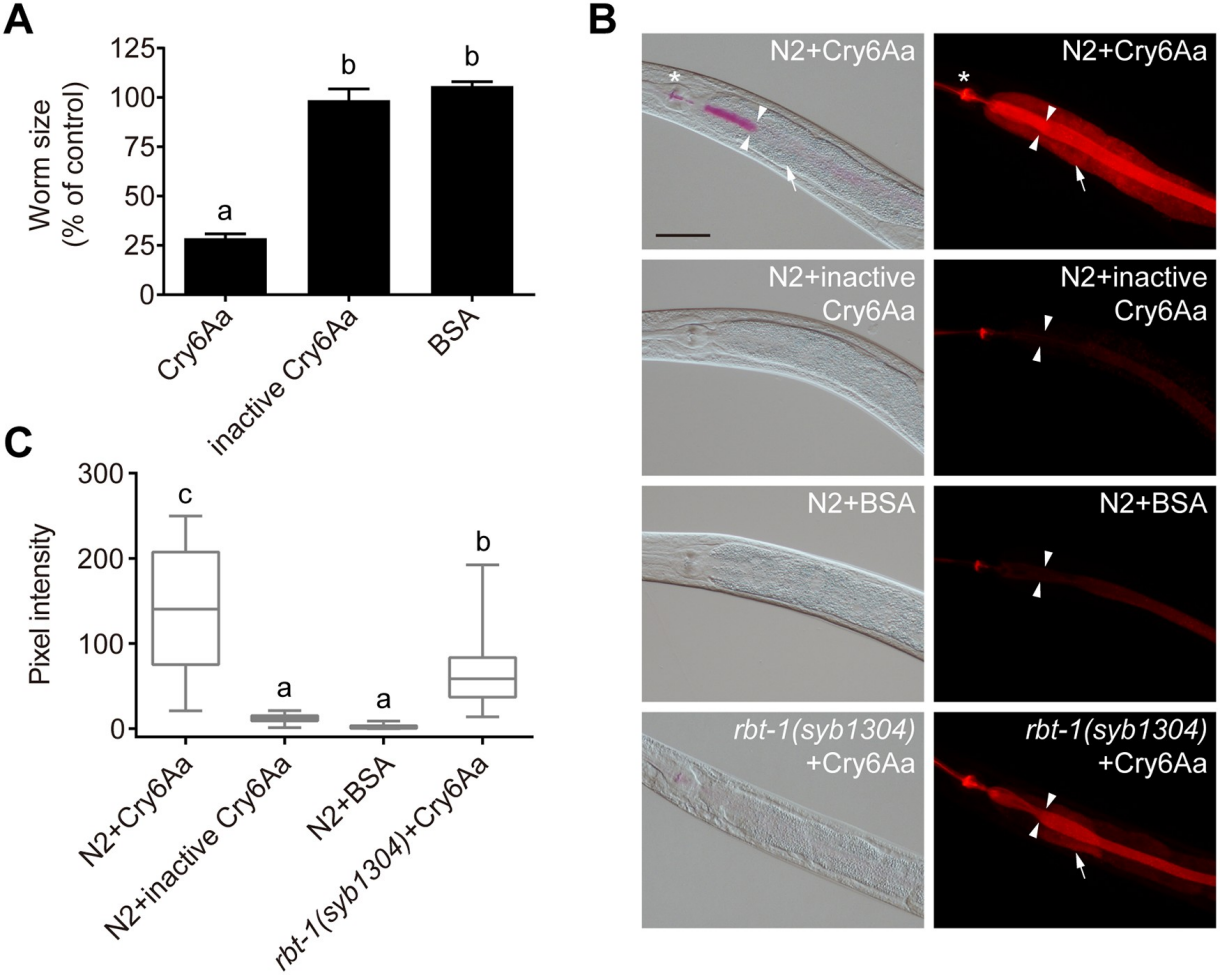
RBT-1 is required for the uptake of Cry6Aa into intestinal cells of *C*. *elegans*. (A) Growth assay was performed using wild-type N2 exposed to rhodamine-labeled Cry6Aa, heat-inactivated rhodamine-labeled Cry6Aa and rhodamine-labeled BSA. Values are means ± SD (*n* = 3 independent trials). Different letters indicate significant differences (*n* = 100 animals; *P* < 0.0001; one-way ANOVA). (B) Cry6Aa diffusion assay. Wild-type N2 were exposed to rhodamine-labeled Cry6Aa, heat-inactivated Cry6Aa and BSA, respectively. Mutant *rbt-1(syb1304)* were exposed to rhodamine-labeled Cry6Aa. Photographs were acquired with DIC (left), and in the rhodamine channel to visualize Cry6Aa or BSA (right). Asterisks indicate pharynx; arrowheads point to intestinal lumen; arrows point to intestinal cells; scale bar, 50 μm. (C) Quantification of pixel intensity of Cry6Aa or BSA in the intestinal cells, parallel to those shown in B. Different letters indicate significant differences (*n* = 50 animals; *P* < 0.001; one-way ANOVA). Data were obtained from three independent experiments.

It was reported that certain glycolipids function as receptors of Cry5Ba, and glycolipid-related genes *bre-2*, *bre-3*, *bre-4* and *bre-5* are required for the uptake of Cry5Ba into intestinal cells of *C*. *elegans*, suggesting that glycolipids are required for Cry5Ba interaction with the plasma membrane of the intestine [27, 39]. To determine whether RBT-1 is required for Cry6Aa to interact with the gut of *C*. *elegans*, we analyzed Cry6Aa internalization by tracing rhodamine-labeled protein. First, we found that rhodamine-labeled Cry6Aa inhibited the growth of N2 and that rhodamine-labeled heat-inactivated Cry6Aa and BSA were nontoxic to animals (Fig 4A). Then, N2 were treated with rhodamine-labeled Cry6Aa, inactivated Cry6Aa and BSA, respectively, for 2 h, and the fluorescence signal was monitored by microscopic observations. We found that Cry6Aa diffused into the intestinal cells of N2, but inactivated Cry6Aa and BSA did not (Fig 4B and 4C). These results indicate that Cry6Aa internalization into gut cells is associated with toxicity. In contrast to wild-type N2, the mutant *rbt-1(syb1304)* and *rbt-1(ttTi3768)* significantly reduced the endocytosis of functional Cry6Aa into gut cells (Fig 4B and 4C and S2 Fig). These results show that RBT-1 is required for the interaction of Cry6Aa toxin with intestinal cells and for its uptake into *C*. *elegans* cells.

### RBT-1 is required for the pore-formation of Cry6Aa in *C*. *elegans*

**Fig 5:**
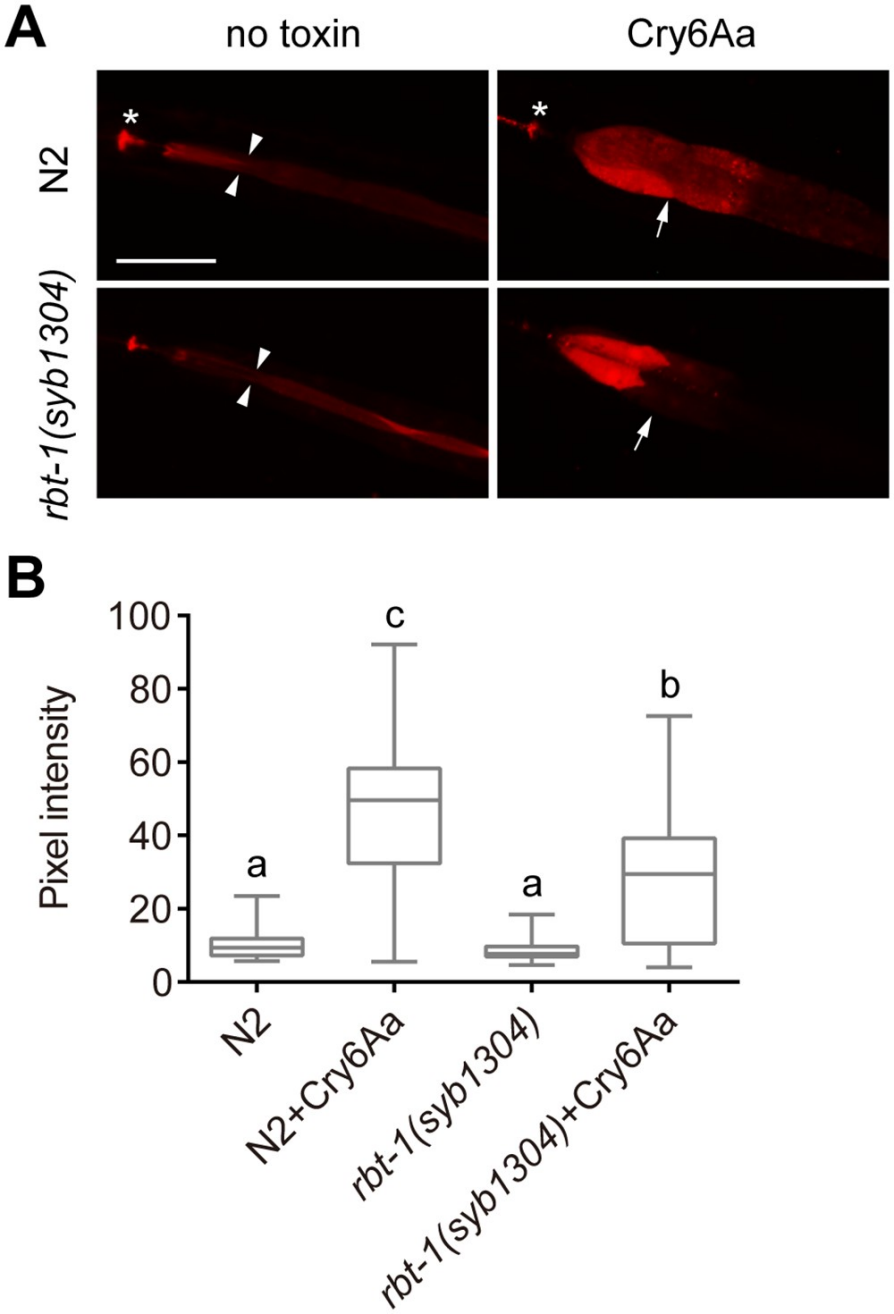
RBT-1 is involved in propidium iodide uptake mediated by Cry6Aa in *C*. *elegans*. (A) Wild-type N2 or mutant *rbt-1(syb1304)* were exposed to 50 μg/ml Cry6Aa or no toxin before propidium iodide (PI) staining, and fluorescence microscopy was used to monitor the signal of PI. Asterisks indicate pharynx; arrowheads point to intestinal lumen; arrows point to intestinal cells; scale bar, 50 μm. (B) Quantification of pixel intensity of PI in the intestinal cells, parallel to those shown in A. Different letters indicate significant differences (*n* = 50 animals; *P* < 0.0001; one-way ANOVA). Data represented three independent experiments.

It has been reported that Cry6Aa is a pore-forming toxin [15] and propidium iodide (PI) can enter cells through pores formed by Cry6Aa in *C*. *elegans* [14]. To determine whether RBT-1 is required for the pore-formation activity of Cry6Aa in nematodes, we conducted PI staining assays. As shown in Fig 5, exposure of Cry6Aa allowed the entry of PI into cells in wild-type nematodes. As expected, the mutation of *rbt-1* significantly reduced the penetration of PI across cell membranes in worms exposed to Cry6Aa (Fig 5 and S3 Fig). Because PI only can permeate cells losing the membrane integrity, these data indicate that RBT-1 participates in the pore-formation activity of Cry6Aa in *C*. *elegans*.

### RBT-1 is unrelated with necrosis

**Fig 6:**
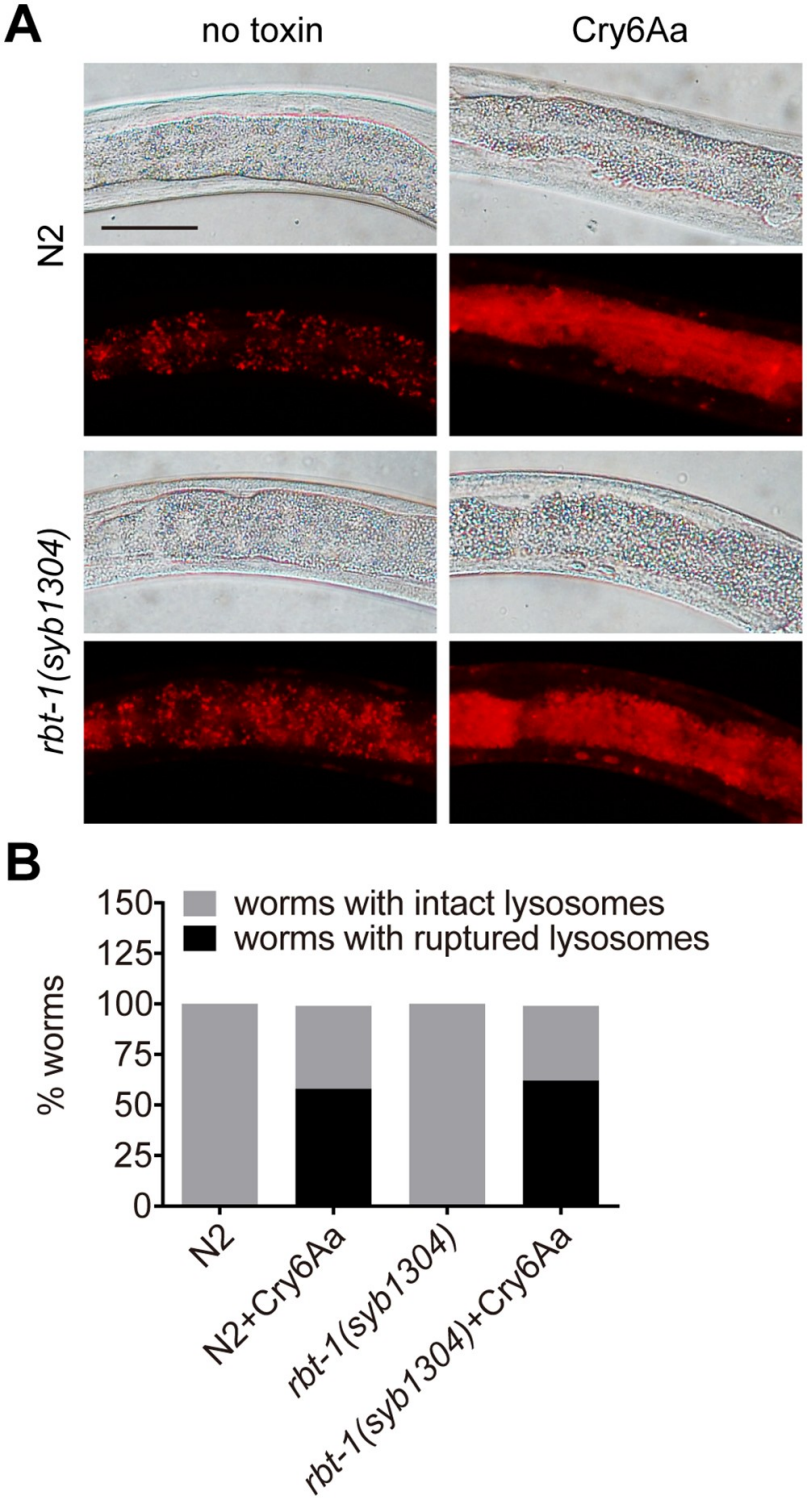
The mutation of *rbt-1* does not affect the lysosomal rupture caused by Cry6Aa in *C*. *elegans*. (A) Wild-type N2 or mutant *rbt-1(syb1304)* were exposed to 50 μg/ml Cry6Aa and stained by Lysotracker. Scale bar, 50 μm. (B) Quantification of worms with lysosomal rupture, parallel to those shown in A. Approximately 50 animals were used for each set of data.

We previously showed that Cry6Aa induces cell death by necrosis signaling pathway in *C*. *elegans*, and that the rupture of lysosomes is a characteristic of necrosis [23]. Here, we found that in the presence of Cry6Aa, mutant *rbt-1(syb1304)* or *rbt-1(ttTi3768)* showed similar destruction of lysosomal integrity to wild-type worms (Fig 6A and S4A Fig). The proportion of N2 with ruptured lysosomes was similar to that of *rbt-1(syb1304)* or *rbt-1(ttTi3768)* (Fig 6B and S4B Fig). These results indicate that RBT-1 is not involved in Cry6Aa-induced nematicidal necrosis.

### RBT-1 does not mediate the toxicity of Cry5Ba to *C*. *elegans*

**Fig 7:**
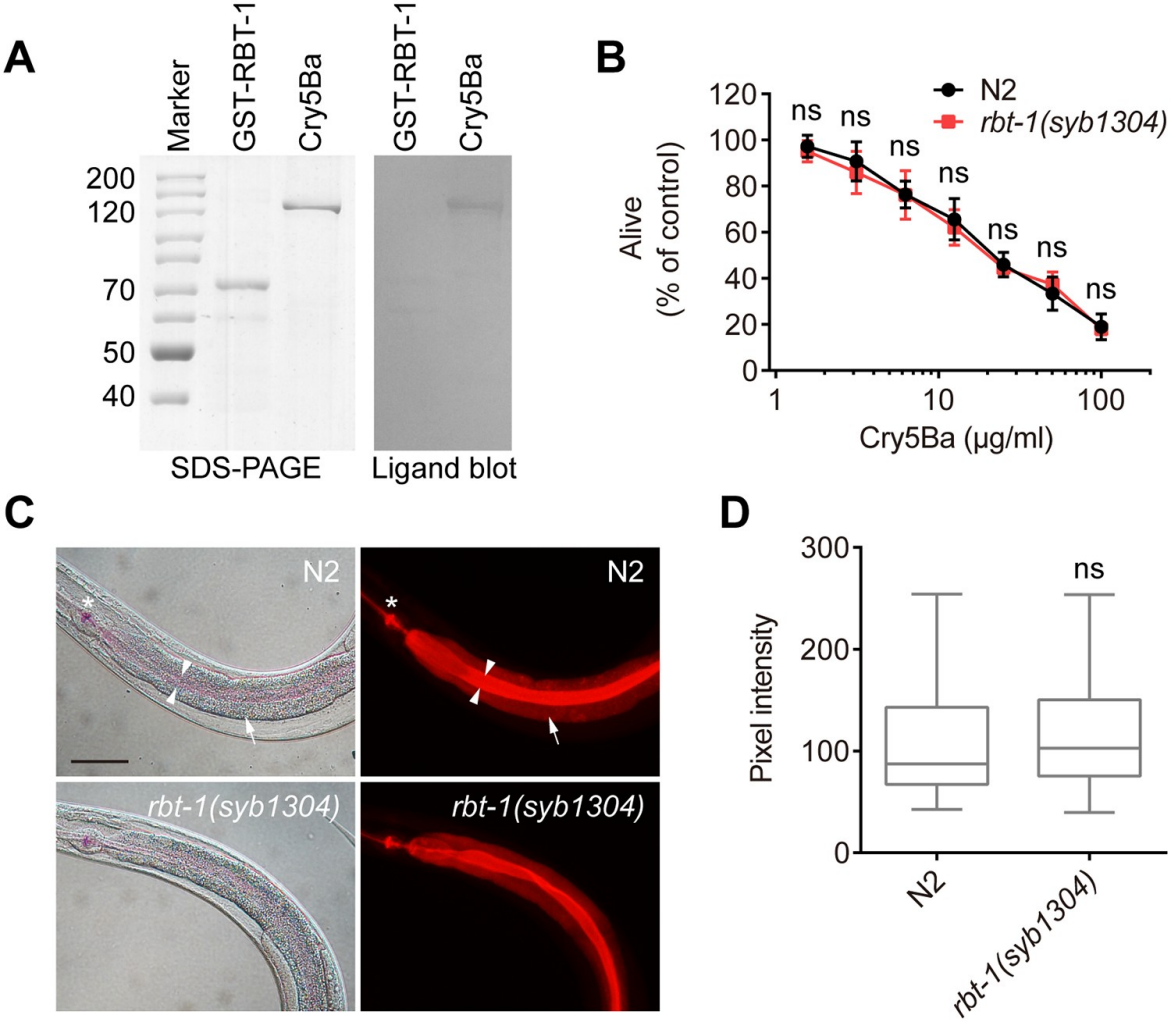
Mutant *rbt-1(syb1304)* is similarly sensitive to Cry5Ba as wild-type N2. (A) Western ligand blot for testing whether Cry5Ba binds to RBT-1. Purified GST-RBT-1 and Cry5Ba were separated on SDS-PAGE and visualized by staining with Coomassie Brilliant Blue R250 (left). An identical gel was electrotransferred to NC membrane, which was incubated with Cry5Ba. After removing unbound Cry5Ba, the membrane was incubated with anti-Cry5Ba antibody (right). (B) Mortality assays of wild-type N2 and mutant *rbt-1(syb1304)* on Cry5Ba. *rbt-1(syb1304)* showed similar susceptibility to Cry5Ba as compared with N2. Values are means ± SD (*n* = 3 independent trials). ns, not significant (*n* = 100 animals; *P* > 0.05; two-way ANOVA). (C) RBT-1 is not required for the internalization of Cry5Ba into intestinal cells in *C*. *elegans*. Both N2 (top) and *rbt-1(syb1304)* (bottom) were treated by rhodamine-labeled Cry5Ba as described above. Photographs were acquired with DIC (left), and in the rhodamine channel to visualize Cry5Ba (right). Asterisks indicate pharynx; arrowheads point to intestinal lumen; arrows point to intestinal cells; scale bar, 50 μm. (D) Quantification of pixel intensity of Cry5Ba in the intestinal cells, parallel to those shown in C. ns, not significant (*n* = 50 animals; *P* > 0.05; two-tailed *t* test). Data were obtained from three independent experiments.

A previous study showed that Cry5B-resistant mutant strains affected in glycolipids synthesis of *C*. *elegans* were sensitive to Cry6Aa [22], so the question was raised whether RBT-1-mediated resistance is specific to Cry6Aa, but not to Cry5Ba. To test this hypothesis, we first carried out western ligand blot and found that Cry5Ba hardly bound to RBT-1 (Fig 7A). Then, we examined the mortality rate of wild-type N2 and mutant *rbt-1(syb1304)* and *rbt-1(ttTi3768)* after treatment with Cry5Ba. Bioassay showed that the sensitivity of mutant *rbt-1(syb1304)* or *rbt-1(ttTi3768)* to Cry5Ba was similar to that of N2 (Fig 7B and S5 Fig). Furthermore, we observed that the internalization of rhodamine-labeled Cry5Ba into gut cells of N2 and *rbt-1(syb1304)* was similar (Fig 7C and 7D). These data indicate that RBT-1 is dispensable for Cry5B toxicity to *C*. *elegans*.

## Discussion

Different Cry proteins exhibit nematicidal activity, such as Cry5Ba and Cry6Aa [24]. Cry6Aa shows no sequence similarity to Cry5Ba nor with any other Cry protein from the three-domain family. Furthermore, Cry5Ba-resistant *C*. *elegans bre* mutants are sensitive to Cry6Aa, and combination of Cry6Aa and Cry5Ba shows synergistic activity against nematodes [21, 22]. These data support that Cry6Aa has a different mode of action compared to Cry5Ba and thus, could be a promising alternative or supplement to Cry5Ba for the biocontrol of plant-pathogenic nematodes.

However, the nematicidal mechanism and specific receptors of Cry6Aa remained unknown. Many known Cry receptors are anchored by GPIs on the host cell surface [8, 30]. In this study, RBT-1 was identified as a Cry6Aa-binding protein in *C*. *elegans*. The fact that RBT-1 was cleaved out by treatment with PI-PLC treatment indicated that RBT-1 is a GPI-anchored protein. Furthermore, bioinformatics analysis suggested that RBT-1 is a GPI-anchored, glycosylated protein found in the gut of *C*. *elegans*. Rao *et al* reported that RBT-1 was identified from the lipid raft fraction of *C*. *elegans* by geLC-MS/MS [40]. Lipid rafts are microdomains of the phospholipid bilayer with GPI-anchored proteins and transmembrane proteins, serving as a platform for protein-protein interactions and protein attachment for transport within the cells [41]. BLAST results indicate that RBT-1 shows no sequence identity to the known receptors for other Cry proteins. The amino acid sequence identity of the Cry6Aa-binding receptor implies a special mode of action of this toxin.

Bacterial toxins target the cell surface through binding interaction with their corresponding receptors, contributing to the specificity of toxins and increasing their effective concentrations in the membrane. In the case of Dipteran mosquito species, *Anopheles gambiae*, CR9-CR11 and CR11-MPED, peptides of the receptor cadherin, bind Cry4B toxin with high affinities of 13 and 23 nM [42]. Furthermore, the affinities of interaction of Cry11Ba with AgALP1 and AgAPN2 are 23.9 nM and 6.4 nM respectively [30, 43]. In *C*. *elegans*, Cry5B specifically bind to glycolipid component B with an apparent dissociation constant of 0.73 ± 0.06 μM [20]. In the present work, ELISA binding assays showed that Cry6Aa bound to RBT-1 with high affinity (~ 21 nM). In addition, the specificity of the interaction between RBT-1 and Cry6Aa was confirmed by the homologous competition binding assay. The identification of RBT-1 as a Cry6Aa binding protein suggested its role as a receptor of this toxin.

Cry toxins bind to their corresponding receptors to exert toxicity. In various insects, mutations of such receptors, as cadherin, aminopeptidase-N and alkaline phosphatase, affected insecticidal activity of Bt Cry proteins to those larvae [4]. Also *C*. *elegans* with glycolipids mutation are resistant to nematicidal Cry5Ba [27]. In the current study, we found that mutants *rbt-1(syb1304)* or *rbt-1(ttTi3768)* showed decreased susceptibility to Cry6Aa as determined by growth and survival rates. Meanwhile, *rbt-1(syb1304)* or *rbt-1(ttTi3768)* mutants were similarly sensitive to Cry5Ba toxin as wild-type N2. These results suggest that RBT-1 is a specific receptor for Cry6Aa, but not for Cry5Ba. However, it is worth noting that the resistance of *rbt-1(syb1304)* or *rbt-1(ttTi3768)* was incomplete. Thus, additional unidentified receptors may also participate in Cry6Aa toxicity.

Receptor-mediated internalization fulfils a critical role in mediating the toxicity of many bacterial toxins, including anthrax toxin [44–46], Shiga toxin [47], cholera toxin (CT) [48], clostridial binary toxins [49], clostridial glucosylating toxins (CGTs) [50], plasmid-encoded toxin (Pet) [51], and so on. In contrast, endocytosis protects hosts from attacks from some other bacterial toxins, such as streptolysin O (SLO) [52], Cry5B [29] and α-toxin [53]. In this study, we demonstrated that RBT-1 was required for the endocytosis of Cry6Aa, indicating that RBT-1 is involved in the interaction of Cry6Aa with *C*. *elegans* gut cell surface. Further experiments are necessary to reveal the relationship between endocytosis and the activity of Cry6Aa, either promoting toxicity or alleviating toxin-caused damage.

Recently, we and other groups identified Cry6Aa as a member of alpha helical pore-forming toxins [13–15]. This study showed that RBT-1 is involved in the Cry6Aa-mediated pore-formation in *C*. *elegans*. In addition, we previously reported that Cry6Aa triggers cell death by necrosis signaling pathway [23]. However, RBT-1 was unrelated to the nematicidal necrosis pathway. Therefore, Cry6Aa might utilize multiple pathways to kill nematodes. On one hand, similar to insecticidal Cry proteins, the pore-formation related to RBT-1 results in the death of nematodes. On the other hand, Cry6Aa kills worms through ASP-1-dependent necrosis pathway. Also, there may be other nematicidal processes triggered by Cry6Aa. Further research is needed to clarify the detailed nematicidal Cry6Aa mode of action.

In conclusion, we revealed that CUB-like-domain containing RBT-1 functions as a receptor for Cry6Aa, representing a new class of receptors for Cry proteins. These findings increase our knowledge of the interaction between Bt Cry proteins and nematodes. Further research is needed to understand the role of RBT-1 in the process of Cry6Aa intoxication. Moreover, identification of other unknown receptors is necessary to shed light on the overall mechanism of action of Cry6Aa.

## Supporting information

S1 Fig Mutant *rbt-1(ttTi3768)* reduced the susceptibility to Cry6Aa compared with N2. *rbt-1(ttTi3768)* is a mutant strain with the insertion of *Mos* transposon in the *rbt-1* gene. Dose-dependent growth assay (A) and mortality assay (B) were performed using Cry6Aa to quantitatively compare the sensitivities of wild-type N2 to mutant *rbt-1(ttTi3768)*. Values are means ± SD (*n* = 3 independent trials). Asterisks represent significant differences (*n* = 100 animals; ***P < 0.001 and ****P < 0.0001; two-way ANOVA).
(TIF)

S2 Fig The uptake of Cry6Aa into intestinal cells is reduced in mutant *rbt-1(ttTi3768)* compared with N2. (A) Cry6Aa diffusion assay. Wild-type N2 and mutant *rbt-1(ttTi3768)* were exposed to rhodamine-labeled Cry6Aa. Photographs were acquired with DIC (left), and in the rhodamine channel to visualize Cry6Aa (right). Asterisks indicate pharynx; arrowheads point to intestinal lumen; arrows point to intestinal cells; scale bar, 50 μm. (B) Quantification of pixel intensity of Cry6Aa in the intestinal cells, parallel to those shown in A. Asterisks indicate significant differences (*n* = 50 animals; ****P < 0.0001; two-tailed *t* test). Data were obtained from three independent experiments.
(TIF)

S3 Fig The Cry6Aa-mediated uptake of propidium iodide is reduced in mutant *rbt-1(ttTi3768)* compared with N2. (A) Wild-type N2 or mutant *rbt-1(ttTi3768)* were exposed to 50 μg/ml Cry6Aa or no toxin before propidium iodide (PI) staining, and fluorescence microscopy was used to monitor the signal of PI. Arrowheads point to intestinal lumen; arrows point to intestinal cells; scale bar, 50 μm. (B) Quantification of pixel intensity of PI in the intestinal cells, parallel to those shown in A. Different letters indicate significant differences (*n* = 50 animals; *P* < 0.01; one-way ANOVA). Data represented three independent experiments.
(TIF)

S4 Fig The mutation of *rbt-1* does not affect the lysosomal rupture caused by Cry6Aa in *C*. *elegans*. (A) Wild-type N2 or mutant *rbt-1(ttTi3768)* were exposed to 50 μg/ml Cry6Aa and stained by Lysotracker. Scale bar, 25 μm. (B) Quantification of worms with lysosomal rupture, parallel to those shown in A. Approximately 50 animals were used for each set of data.
(TIF)

S5 Fig Mortality assays of wild-type N2 and mutant *rbt-1(ttTi3768)* after treatment with Cry5Ba. *rbt-1(ttTi3768)* showed similar susceptibility to Cry5Ba as compared with N2. Values are means ± SD (*n* = 3 independent trials). ns, not significant (*n* = 100 animals; *P* > 0.05; two-way ANOVA).
(TIF)
