# Supplementary figures and images for: The Caenorhabditis elegans CUB-like-domain containing protein RBT-1 functions as a receptor for Bacillus thuringiensis Cry6Aa toxin
Source: PLoS Pathog. 2020 May 5;16(5):e1008501. doi: 10.1371/journal.ppat.1008501 (PMC7228132; doi:10.1371/journal.ppat.1008501)

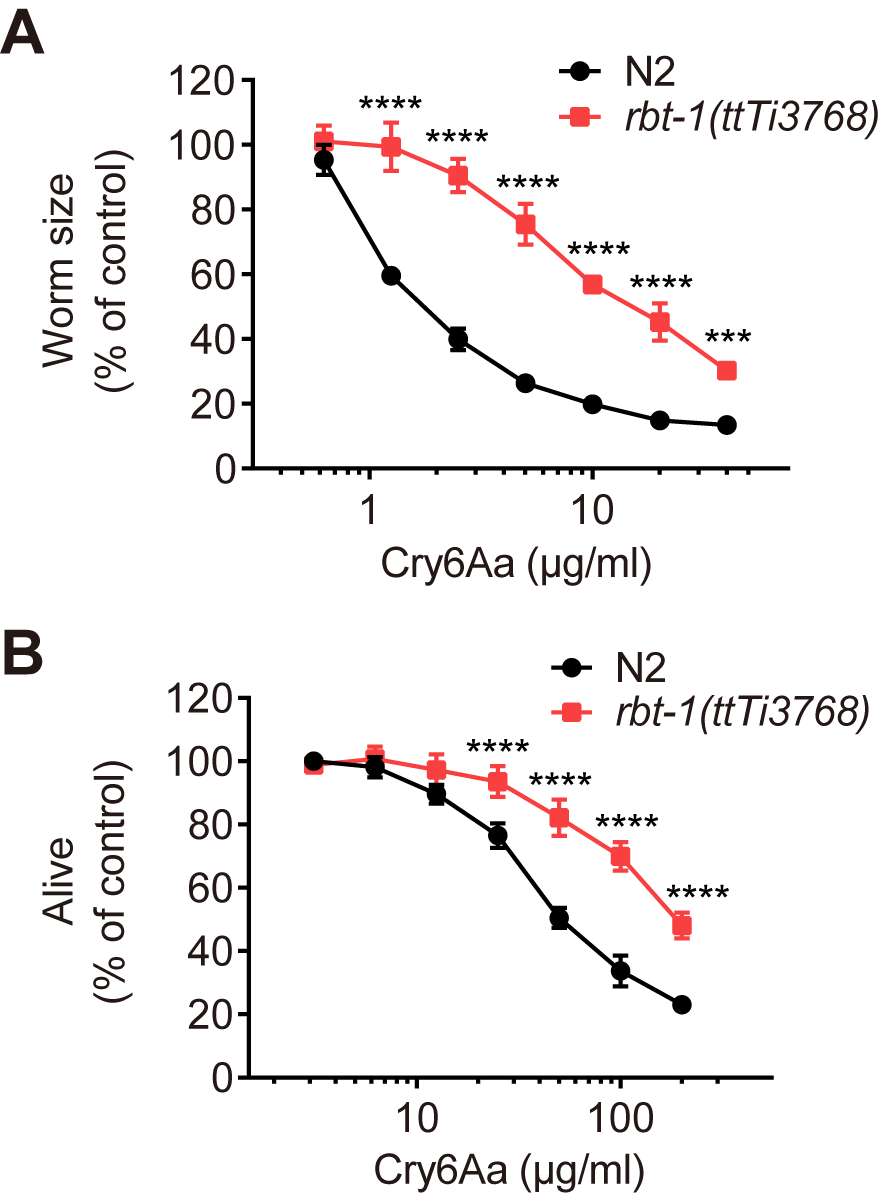

Supplement: S1 Fig — rbt-1(ttTi3768) is a mutant strain with the insertion of Mos transposon in the rbt-1 gene. Dose-dependent growth assay (A) and mortality assay (B) were performed using Cry6Aa to quantitatively compare the sensitivities of wild-type N2 to mutant rbt-1(ttTi3768). Values are means ± SD (n = 3 independent trials). Asterisks represent significant differences (n = 100 animals; ***P < 0.001 and ****P < 0.0001; two-way ANOVA). (TIF) [file ppat.1008501.s001.tif]

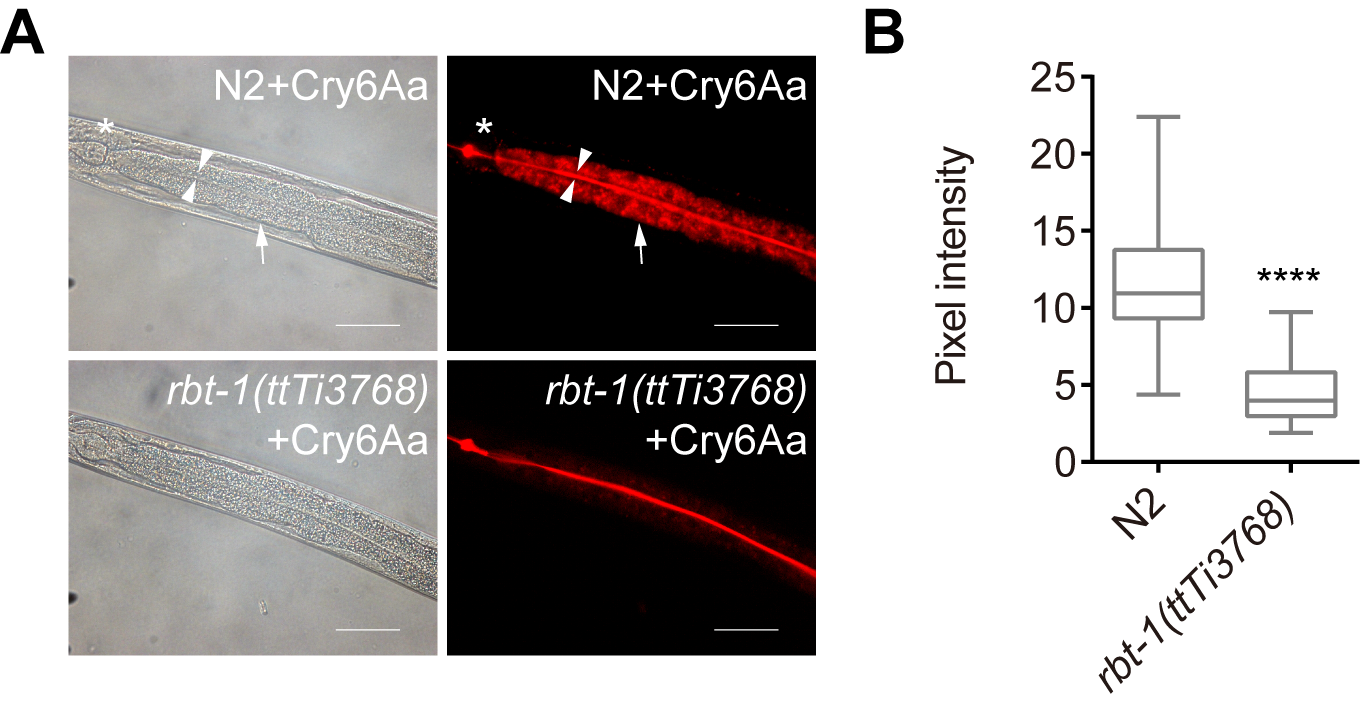

Supplement: S2 Fig — (A) Cry6Aa diffusion assay. Wild-type N2 and mutant rbt-1(ttTi3768) were exposed to rhodamine-labeled Cry6Aa. Photographs were acquired with DIC (left), and in the rhodamine channel to visualize Cry6Aa (right). Asterisks indicate pharynx; arrowheads point to intestinal lumen; arrows point to intestinal cells; scale bar, 50 μm. (B) Quantification of pixel intensity of Cry6Aa in the intestinal cells, parallel to those shown in A. Asterisks indicate significant differences (n = 50 animals; ****P < 0.0001; two-tailed t test). Data were obtained from three independent experiments. (TIF) [file ppat.1008501.s002.tif]

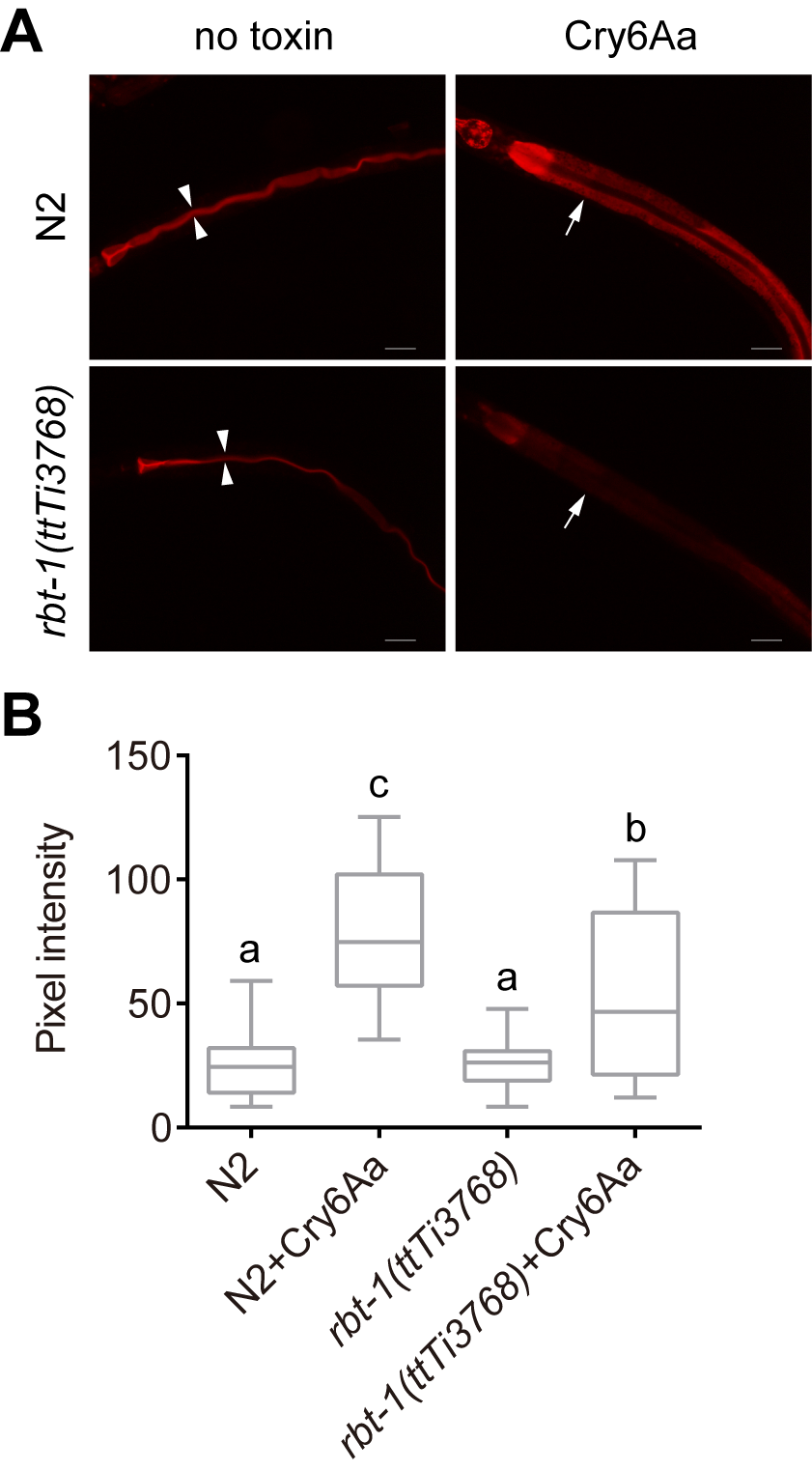

Supplement: S3 Fig — (A) Wild-type N2 or mutant rbt-1(ttTi3768) were exposed to 50 μg/ml Cry6Aa or no toxin before propidium iodide (PI) staining, and fluorescence microscopy was used to monitor the signal of PI. Arrowheads point to intestinal lumen; arrows point to intestinal cells; scale bar, 50 μm. (B) Quantification of pixel intensity of PI in the intestinal cells, parallel to those shown in A. Different letters indicate significant differences (n = 50 animals; P < 0.01; one-way ANOVA). Data represented three independent experiments. (TIF) [file ppat.1008501.s003.tif]

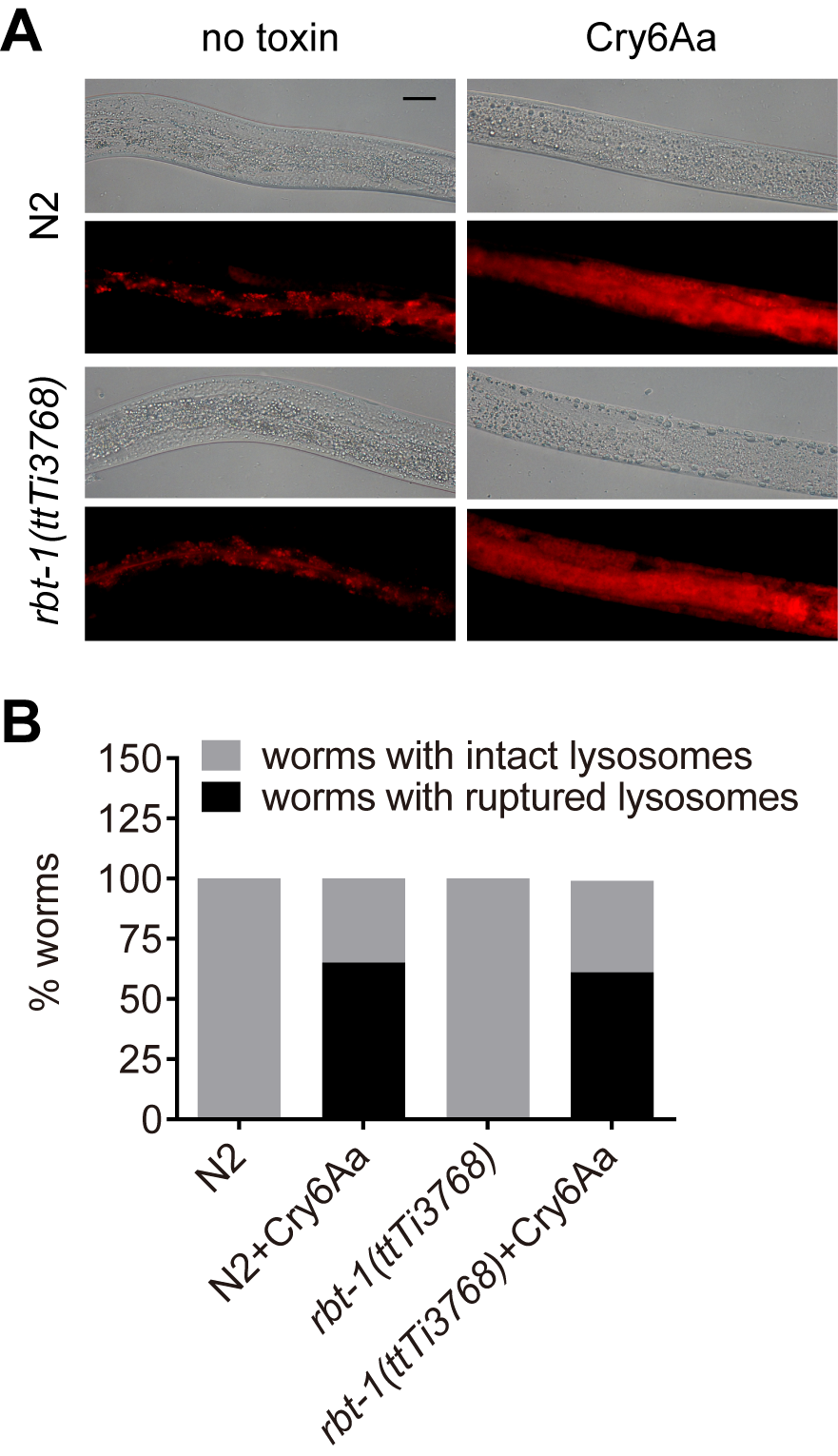

Supplement: S4 Fig — (A) Wild-type N2 or mutant rbt-1(ttTi3768) were exposed to 50 μg/ml Cry6Aa and stained by Lysotracker. Scale bar, 25 μm. (B) Quantification of worms with lysosomal rupture, parallel to those shown in A. Approximately 50 animals were used for each set of data. (TIF) [file ppat.1008501.s004.tif]

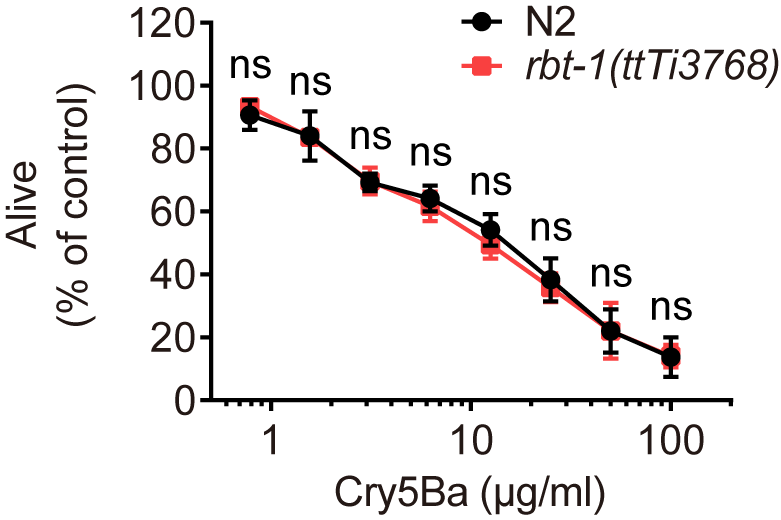

Supplement: S5 Fig — rbt-1(ttTi3768) showed similar susceptibility to Cry5Ba as compared with N2. Values are means ± SD (n = 3 independent trials). ns, not significant (n = 100 animals; P > 0.05; two-way ANOVA). (TIF) [file ppat.1008501.s005.tif]
